# Supplementary material for: Human-induced climate change amplification on storm dynamics in Valencia’s 2024 catastrophic flash flood
Source: Nat Commun. 2026 Feb 17;17:1492. doi: 10.1038/s41467-026-68929-9 (PMC12913788; doi:10.1038/s41467-026-68929-9)
Supplement: Supplementary file 1 — Supplementary information [file 41467_2026_68929_MOESM1_ESM.pdf]

Supplementary Information for

Human-induced climate change amplification on storm dynamics in Valencia's 2024 catastrophic flash flood

C. Calvo-Sancho[1,2]*, J. Díaz-Fernández[1], J.J. González-Alemán[3], A. Halifa-Marín[4,5], M.M. Miglietta[6], C. Azorin-Molina[2], A. F. Prein[7], A. Montoro-Mendoza[1,8], P. Bolgiani[9], A. Morata[3], M.L. Martín[1,10]

[1] Department of Applied Mathematics. Faculty of Computer Engineering, Universidad de Valladolid, Spain.

[2] Centro de Investigaciones sobre Desertificación, Consejo Superior de Investigaciones Científicas (CIDE, CSIC-UV-GVA), Climate, Atmosphere and Ocean Laboratory (Climatoc-Lab), Moncada, Valencia, Spain.

[3] Spanish State Meteorological Agency (AEMET), Department of Science, Spain.

[4] Instituto Pirenaico de Ecología, Consejo Superior de Investigaciones Científicas (IPE-CSIC), 50059 – Zaragoza, Spain.

[5] Laboratorio de Climatología y Servicios Climáticos (LCSC), CSIC-Universidad de Zaragoza, Spain

[6] National Research Council of Italy, Institute of Atmospheric Sciences and Climate (CNR-ISAC), Padua, Italy.

[7] Institute of Atmospheric and Climate Science, ETH Zurich, Zurich, Switzerland

[8] Consejo Superior de Investigaciones Científicas (CSIC). Instituto de Geociencias (IGEO), Madrid, España.

[9] Department of Earth Physics and Astrophysics, Faculty of Physics, Complutense University of Madrid, Madrid, Spain.

[10] Interdisciplinary Mathematics Institute. Universidad Complutense de Madrid, Spain.

* Corresponding Author: Carlos Calvo-Sancho (carlos.calvo.sancho@uva.es)

Supplementary Table 1: CMIP6 models. Further details for each model are available in the references listed in this table.

GCM	Institute ID	Resolution (lat. x lon.)	Reference
**AWI-CM-1-1-MR**	AWI (Germany)	0.937° x 0.937°	Semmler et al. (2018)
**CESM2-WACCM**	NCAR (USA)	1.3° x 0.9°	Danabasoglu (2019)
**EC-Earth3**	EC-Earth-Consortium (Europe)	0.7° x 0.7°	EC-Earth (2019)
**FGOALS-g3**	CAS (China)	2° x 2°	Li et al. (2020)
**GISS-E2-1-G**	NASA (USA)	2° x 2.5°	Kelley et al. (2020)
**GISS-E2-1-H**	NASA (USA)	2° x 2.5°	Kelley et al. (2020)
**INM-CM4-8**	INM (Russia)	1.5° x 2°	Volodin et al. (2018)
**INM-CM5-0**	INM (Russia)	1.5° x 2°	Volodin et al. (2018)
**IPSL-CM6A-LR**	IPSL (France)	1.3 x 2.5°	Boucher et al. (2018)
**MCM-UA-1-0**	UA (USA)	2.25° x 3.75°	Stouffer (2019)
**MIROC-ES2L**	MIROC (Japan)	2.8° x 2.8°	Tachiiri et al. (2019)
**MIROC6**	MIROC (Japan)	1.4° x 1.4°	Tatebe et al. (2019)
**MRI-ESM2-0**	MRI (Japan)	1.121° x 1.125°	Yukimoto et al. (2019)
**NorESM2-LM**	NCC (Norway)	1.9° x 2.5°	Seland et al. (2020)
**NorESM2-MM**	NCC (Norway)	0.94° x 1.25°	Seland et al. (2020)

Supplementary Table 2. Percentage increase in precipitation gridpoint weighted average and area for the factual simulation compared to counterfactual GCMs. "MAX" indicates that the respective models fail to resolve the specified precipitation threshold under counterfactual conditions.

GCM	Average				Area				Gridpoint weighted average			
	> 100 mm	> 180 mm	> 200 mm	> 300 mm	> 100 mm	> 180 mm	> 200 mm	> 300 mm	> 100 mm	> 180 mm	> 200 mm	> 300 mm
**AWI-CM-1-1-MR**	5	8	8	5	26	25	27	111	33	35	37	121
**CESM2-WACCM**	20	21	20	13	18	71	101	1220	42	107	141	1390
**EC-Earth3**	28	40	33	MAX	48	322	1306	MAX	90	491	1773	MAX
**FGOALS-g3**	9	24	25	11	19	11	29	3758	29	38	60	4182
**GISS-E2-1-G**	33	32	29	MAX	24	526	864	MAX	65	724	1143	MAX
**GISS-E2-1-H**	13	16	13	11	21	46	75	412	37	69	98	469
**INM-CM4-8**	-8	-5	-4	-2	35	9	4	-13	25	3	0	-15
**INM-CM5-0**	19	35	31	MAX	39	73	254	MAX	66	134	364	MAX
**IPSL-CM6A-LR**	11	9	8	3	30	60	69	193	45	75	83	202
**MCM-UA-1-0**	25	34	30	MAX	25	131	353	MAX	57	210	490	MAX
**MIROC-ES2L**	21	36	31	MAX	82	150	456	MAX	120	239	628	MAX
**MIROC6**	18	23	23	MAX	2	37	59	MAX	21	68	95	MAX
**MRI-ESM2-0**	14	33	31	MAX	47	71	179	MAX	68	127	266	MAX
**NorESM2-LM**	5	5	4	5	38	44	48	85	45	50	54	94
**NorESM2-MM**	-2	5	4	8	27	5	8	29	25	10	13	39
**ENSMEAN**	14	30	30	MAX	47	50	105	MAX	68	96	166	MAX

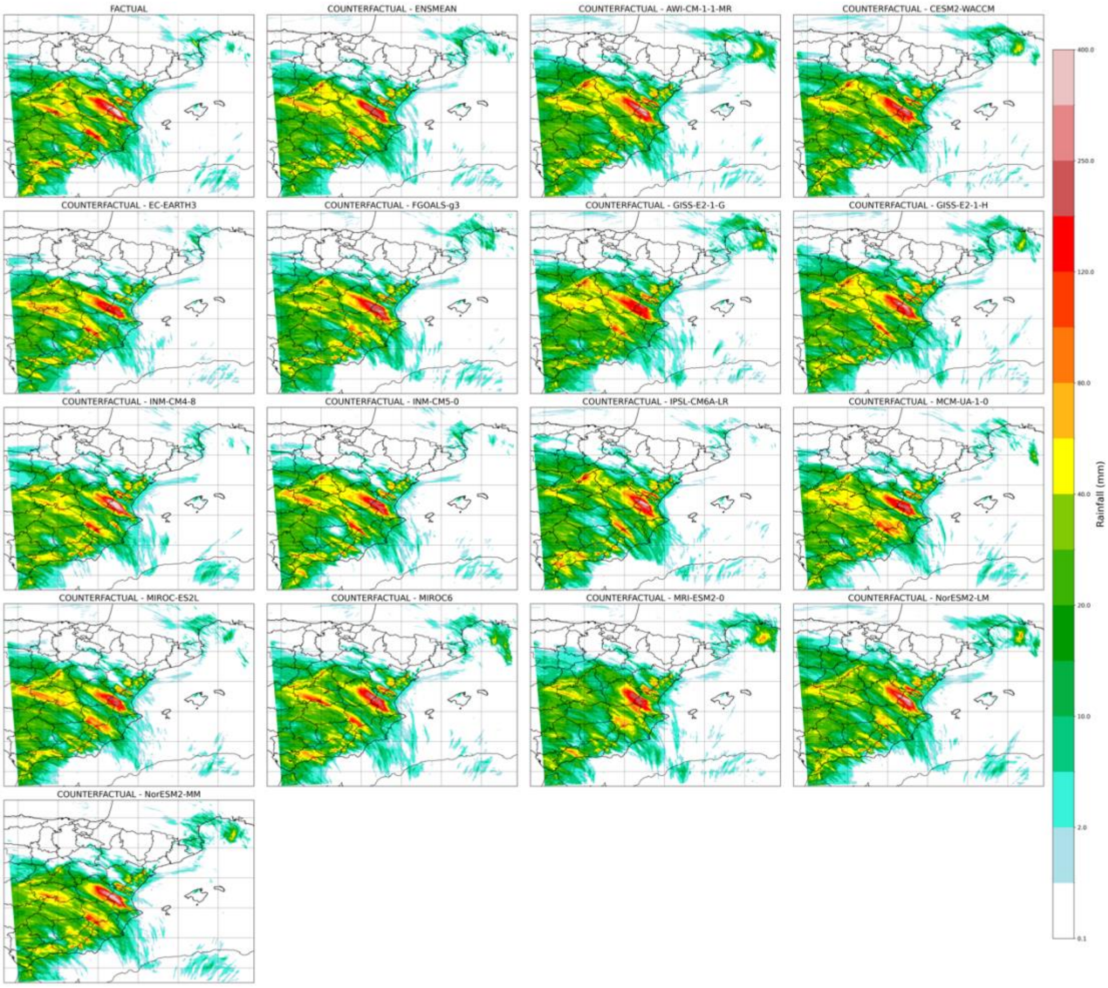

Supplementary Figure 1: Total rainfall accumulation for October 29th for the factual simulation (first plot from the top left), the counterfactual ensemble mean simulation (second plot) and the counterfactual simulations for each climate model forcing (from the third to the last plot).

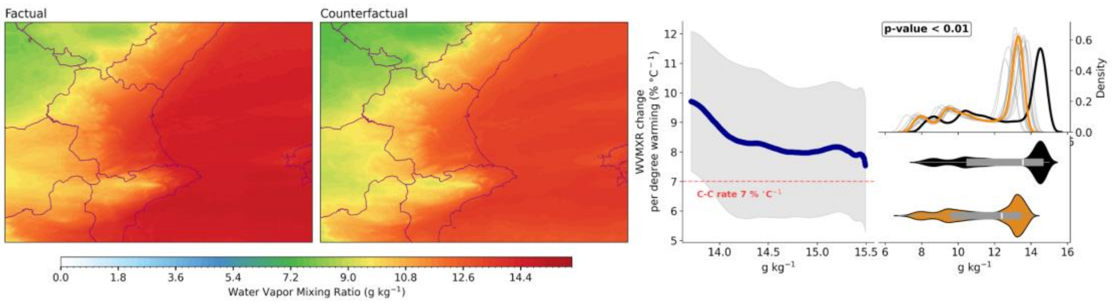

Supplementary Figure 2: Comparison between the factual and counterfactual simulations during the storm period: temporal mean and maximum value within the vertical of the water vapor mixing ratio**.** Adjacent plots show their percentage change

per degree warming (the difference between the two climates states is +1.08ºC), the Probability Density Function for each simulation (black: factual simulation; orange: counterfactual ensemble mean simulation; grey: each counterfactual simulation non-climate CMIP6 model forcing), and the violin plot. The legend in PDF shows statistical significance according to the Mann-Whitney U test.

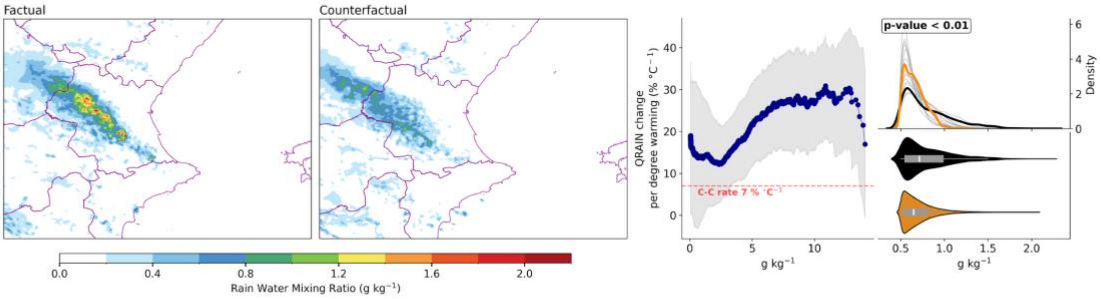

Supplementary Figure 3: As in Extended Data Figure 2, but for rainwater mixing ratio.

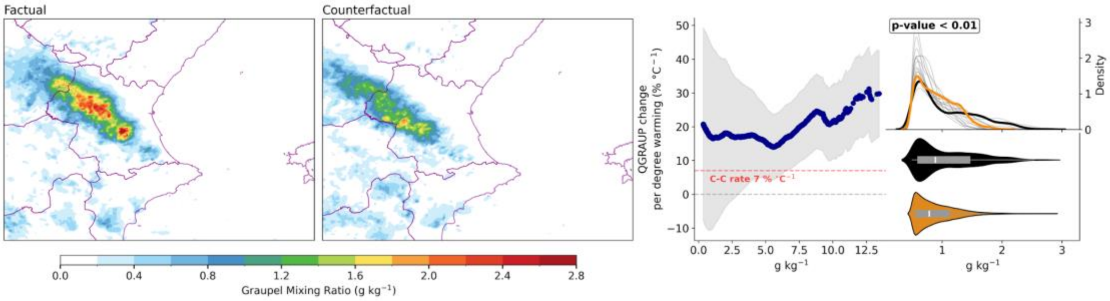

Supplementary Figure 4: As in Extended Data Figure 2, but for graupel mixing ratio.

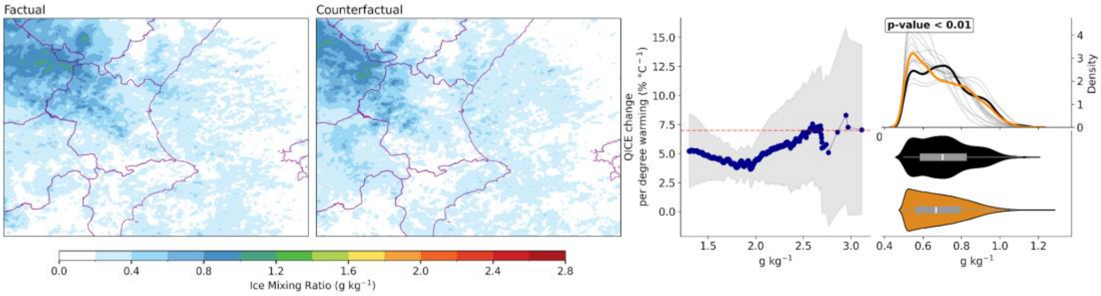

Supplementary Figure 5: As in Extended Data Figure 2, but for ice mixing ratio.

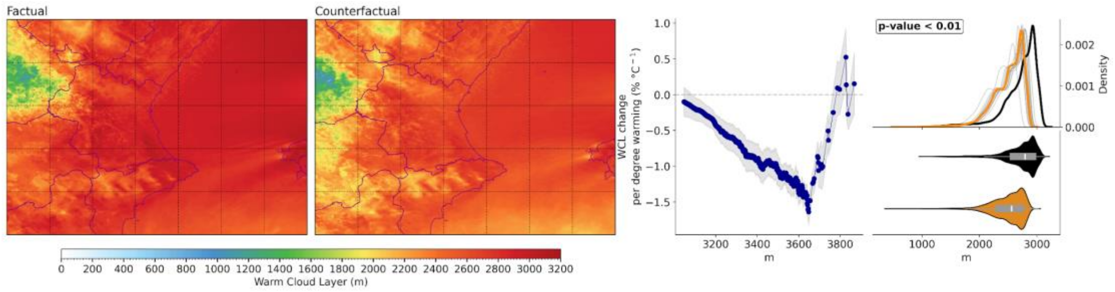

Supplementary Figure 6: As in Extended Data Figure 2, but for warm cloud layer.

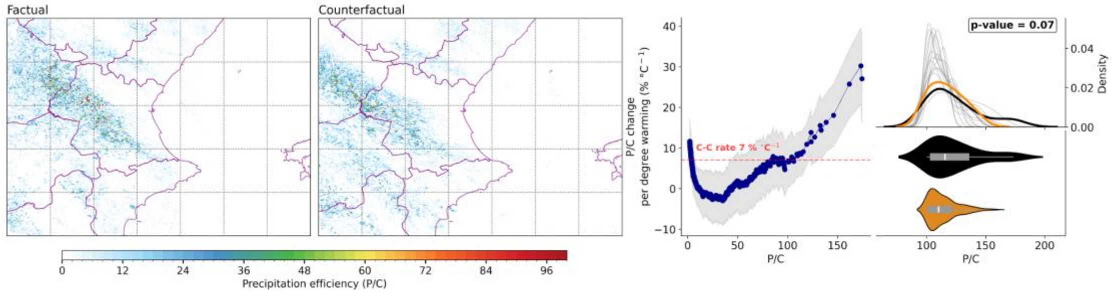

Supplementary Figure 7: As in Extended Data Figure 2, but for precipitation efficiency (P/C).

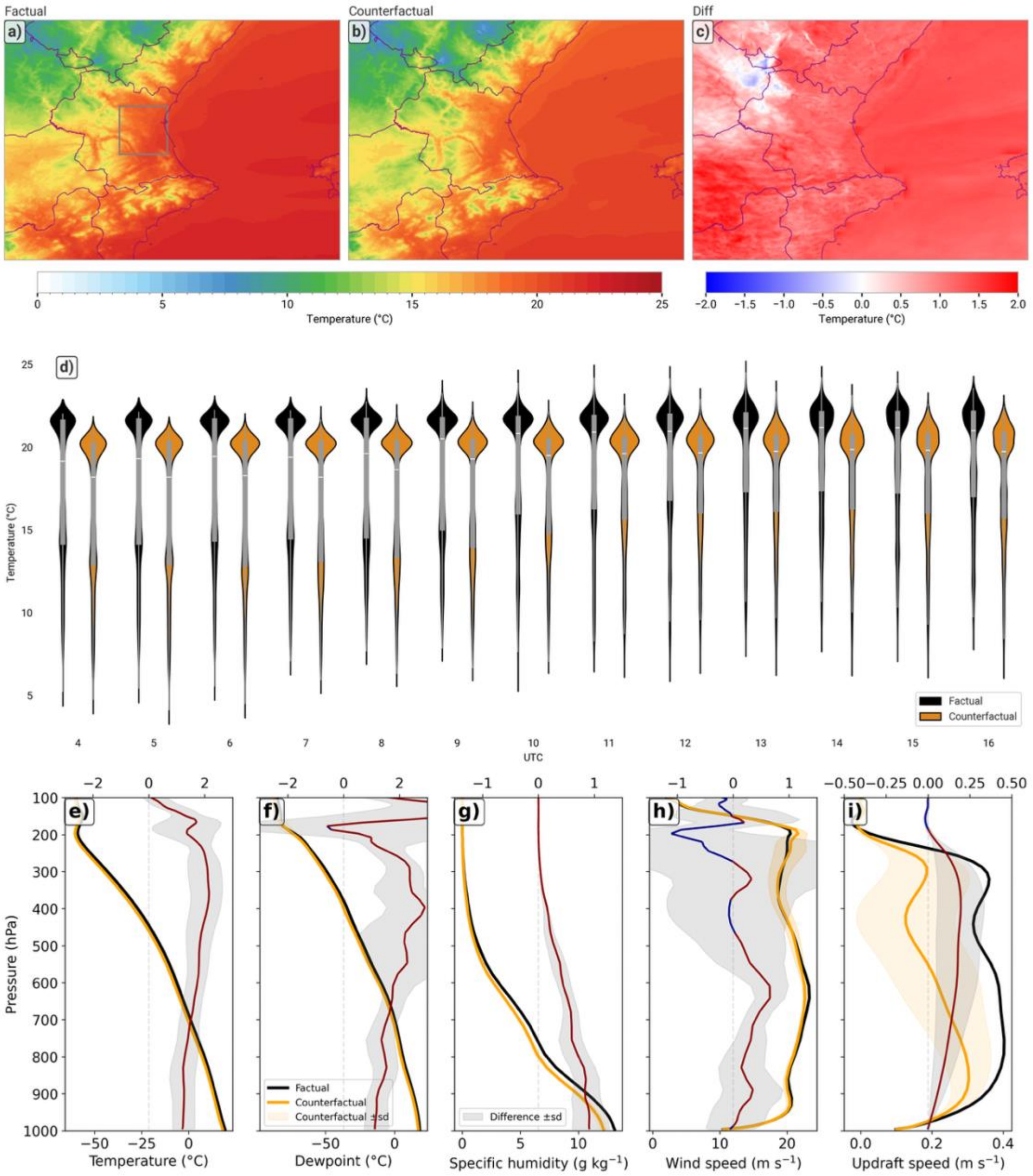

Supplementary Figure 8: 2-m temperature spatial distribution during the storm period in (a) factual, (b) counterfactual runs, and (c) the difference between (a) and (b). (d) 2-m temporal evolution in factual and counterfactuals simulations. Vertical profiles of (e) temperature, (f) dewpoint, (g) specific humidity, (h) wind speed, and (i) updraft speed for factual (black line) and counterfactual (orange line) and their difference. Orange dashed represents ± standard deviation of the 15 counterfactual simulations and grey dashed represents ± standard deviation of the 15 differences between factual and counterfactual. The scale for the difference is reported on the top of the figure.

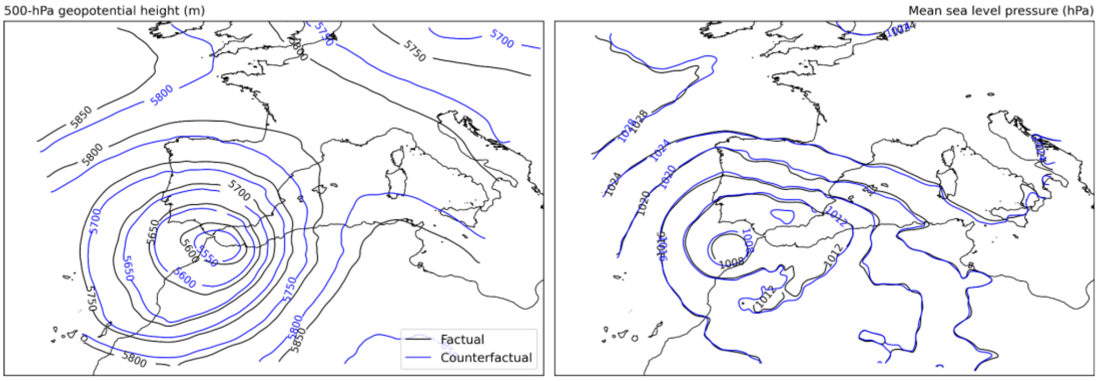

Supplementary Figure 9: Comparison between factual and counterfactual simulation of (a) 500-hPa geopotential height (m) and mean sea level pressure (hPa) at October 29th, 2024 12 UTC.

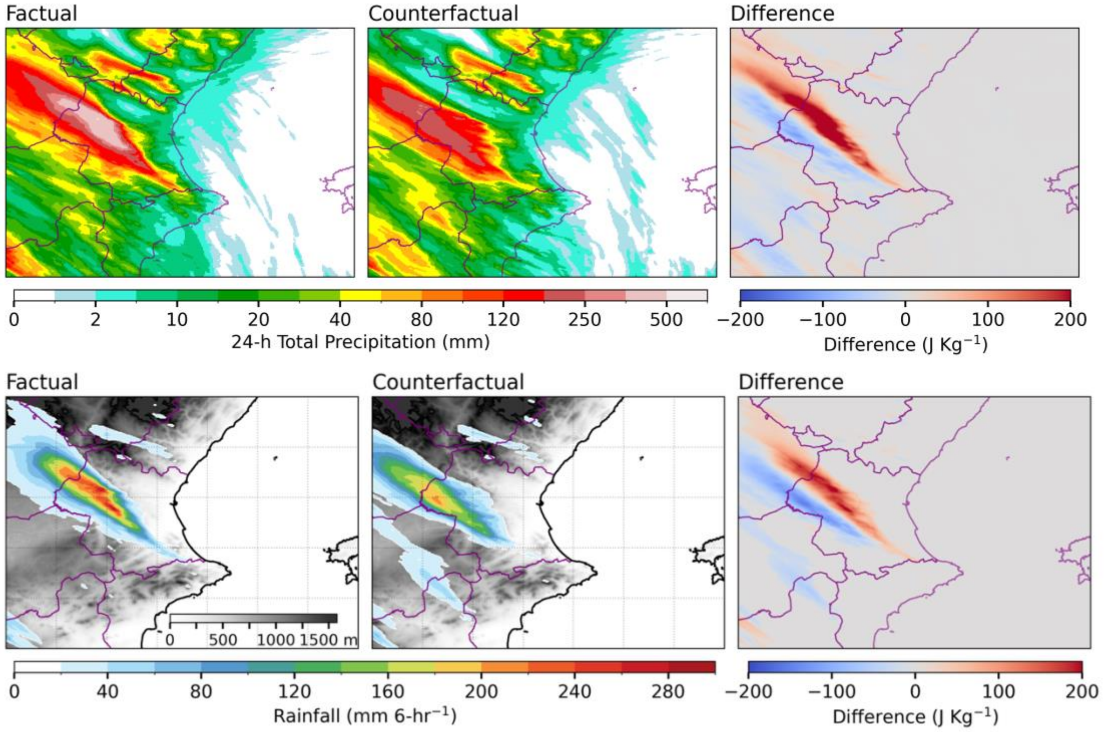

Supplementary Figure 10: Factual (left), counterfactual (center) and difference of Factual and Counterfactual (right) for 24-h total precipitation (above) and 6-h rainfall (bottom).

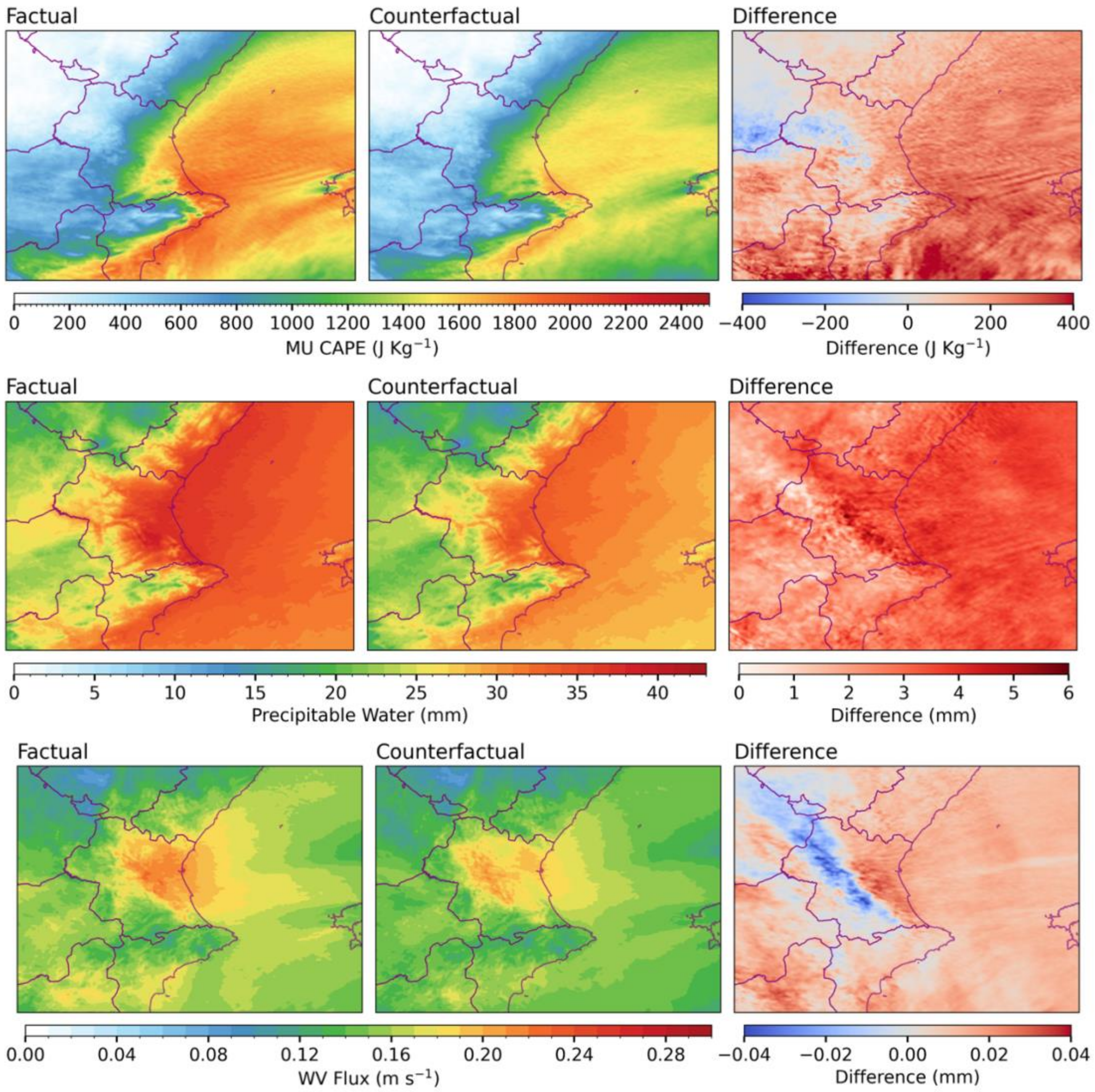

Supplementary Figure 11: As Supplementary Figure 1, but for MU CAPE (above), Precipitable Water (center) and Water Vapor Flux (bottom).

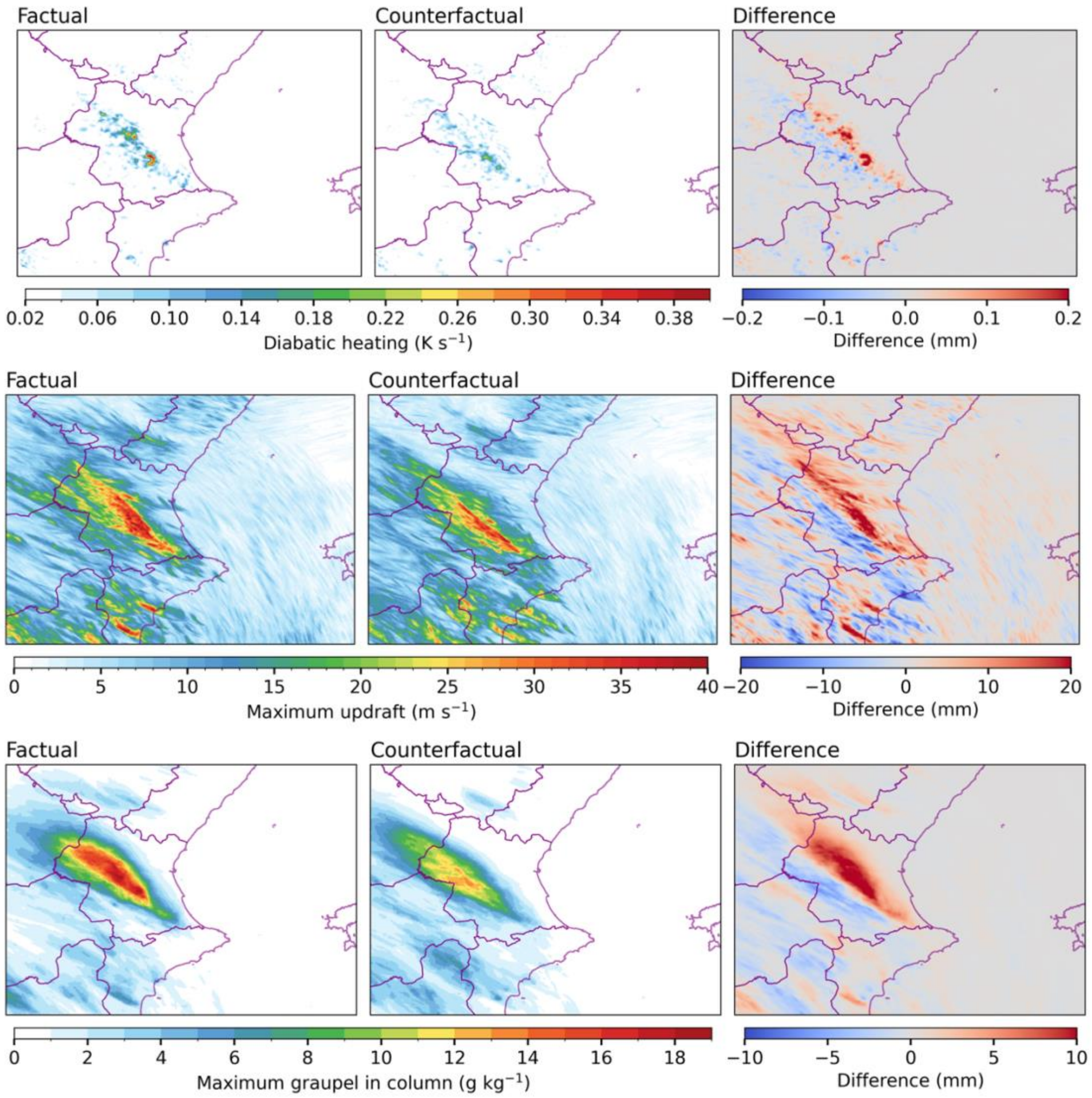

Supplementary Figure 12: As Supplementary Figure 1, but for Diabatic Heating (above), Maximum updraft (center) and Maximum graupel in column (bottom).
